# A Case Report of Suspected Malignant Hyperthermia: How Will the Diagnosis Affect a Patient's Insurability?

**DOI:** 10.1155/2018/6532821

**Published:** 2018-10-30

**Authors:** Brian M. Osman, Isabela C. Saba, William A. Watson

**Affiliations:** ^1^University of Miami, Miller School of Medicine, Department of Anesthesiology, 1400 NW 12^th^ Avenue, Suite 3075, Miami, FL 33136, USA; ^2^University of Miami, Miller School of Medicine, Department of Anesthesiology, 1801 NW 9^th^ Avenue, 5^th^ Floor, Miami, FL 33136, USA; ^3^University of Miami, Miller School of Medicine, Department of Anesthesiology, 1611 NW 12^th^ Avenue, Suite C-300, Miami, FL 33136, USA

## Abstract

The purpose of this case report is to increase awareness that a diagnosis of malignant hyperthermia may have long-lasting or permanent effects on a patient's insurance eligibility or premiums despite legislation providing varying levels of protection from preexisting conditions or genetic discrimination. We present a case of severe rigors, unexplained severe metabolic acidosis, and severe hyperthermia in a patient after general anesthesia for extensive head and neck surgery. The patient was treated for malignant hyperthermia and demonstrated a significant clinical improvement with the administration of dantrolene. Even with an “almost certain” diagnosis of malignant hyperthermia by clinical presentation, genetic testing was negative and the gold-standard caffeine-halothane contracture test has yet to be performed. Laboratory results, clinical grading scales, and genetic testing support a diagnosis of malignant hyperthermia but the gold standard is a live muscle biopsy and caffeine-halothane contracture test. A clinical diagnosis of MH or a positive caffeine-halothane contracture test could result in exclusion from genetic discrimination legislature due to the fact that diagnosis can be confirmed without genetic testing. The fate of the Affordable Care Act may also affect how insurance companies scrutinize this disease. Improving accuracy of MH diagnosis in hospital discharge records will be crucial.

## 1. Introduction

Malignant hyperthermia (MH) is a disease of pharmacogenetics which presents with an abnormal increase in the body's basal metabolic rate, usually after exposure to specific triggering agents such as volatile anesthetic gasses, depolarizing muscle relaxants, and rarely stressors such as heat and vigorous exercise [1]. The Malignant Hyperthermia Association of the United States (MHAUS) estimates that MH affects roughly 1 in 100,000 adult surgeries and 1 in 30,000 pediatric cases [2]. The gold standard for diagnosing MH involves a caffeine-halothane contracture test (CHCT) on a live muscle biopsy sample, but certain clinical diagnostic criteria, laboratory results, and genetic tests may also provide evidence of the diagnosis [3]. MH is difficult and often time-consuming to diagnose but, in the face of a developing crisis, clinicians cannot wait on genetic testing or a CHCT to guide dantrolene treatment. We present a challenging case of a patient whom we treated as if she were experiencing an acute MH crisis after suddenly presenting with severe postoperative rigors, extreme hyperpyrexia, and an unexplained severe metabolic acidosis. While the confirmation of the diagnosis is still pending, the patient's medical record mentions a high suspicion of MH susceptibility and subsequent treatment with dantrolene. The inability to rapidly confirm a diagnosis or even perform the gold-standard biopsy test in a single hospital admission can result in suspected or inaccurately diagnosed cases ending up as a preexisting condition in a patient's permanent medical record [4]. Denial of insurance coverage, exorbitant premiums, and discrimination based on preexisting conditions has garnered enough attention to result in various legislative protections, but the risk of repeal of current policies and implementation of new healthcare campaigns may have profound effects on the affordability or eligibility of insurance for patients who carry the diagnosis of MH. 

## 2. Case Presentation

Informed consent was obtained from the patient as well as authorization to use or disclose protected health information. A 48-year-old ASA III female with infiltrative squamous cell carcinoma in the floor of the mouth presented for an extensive composite resection, free flap reconstruction, neck dissection, and tracheostomy. She had a medical history significant for hypertension, anemia, and 55 pack-years of cigarette smoking. Surgical history included minor procedures and was negative for any anesthetic complications, and there was no reported personal or family history of problems with anesthesia or intolerance to exercise or heat. The patient had no known drug allergies and was taking 20 mg of lisinopril daily, 120 mg of verapamil daily, 5mg of oxycodone as needed for pain, transdermal 50 mcg/hr fentanyl patch, and multivitamins. The anesthetic plan included general anesthesia and a postinduction radial arterial line. Intravenous induction was performed with 100 mcg of fentanyl, 80 mg of lidocaine, 150 mg of propofol, and 30 mg of rocuronium and the patient was intubated with a 7.0 mm endotracheal tube. Anesthesia was maintained with 2% Sevoflurane gas and IV fentanyl for the remainder of the 13-hour surgery. Two units of appropriately typed and crossed packed red blood cells were administered during the case to correct anemia. The arterial blood gases drawn before the conclusion of the case were within acceptable limits (Table 1). The patient emerged from anesthesia without incident, followed commands, denied pain or feeling cold, and was breathing spontaneously through the new tracheostomy. At 22:30 hours, report was given to the Intensivist and vital signs were reported to be stable with a heart rate of 110 beats per minute, blood pressure of 135/75 mmHg, respiratory rate of 22 breaths per minute, oxygen saturation of 100%, and a temperature of 96.4 F. At 23:00 hours, the anesthesia team was called by the ICU to reevaluate the patient for the sudden onset of severe rigors and an acute rise in temperature to 102.5 F, which had occurred over the course of 15 minutes (indicating an increase of 2 degrees F every 5 minutes). An arterial-blood gas taken less than 10 minutes after the onset of symptoms demonstrated a new onset metabolic acidosis and extreme base deficit (Table 2). We observed severe generalized rigors, hyperthermia, tachycardia, and a respiratory rate of 50 breaths per minute. Ruling out the differential diagnoses of uncontrolled pain, sepsis, hypoglycemia, seizure disorder, thyroid storm, neuroleptic malignant syndrome, transfusion reaction, or medication withdrawal, we suspected a diagnosis of MH and called the MHAUS 24-hour MH Hotline. Ice packs were applied to cool the patient and we ordered the appropriate labs to support the MH diagnosis and rule out other conditions (Tables 2 and 3). Within approximately 20 minutes after the initial onset of symptoms, the MH cart was opened and dantrolene (20 mg vials reconstituted with 60 ml of sterile water) was given according to the MHAUS dosing recommendations (2.5mg/kg, followed by 1 mg/kg, and then 1 mg/kg IV every 6 hours until symptoms subside). After the second dose, within approximately 5 minutes, the patient's temperature trended down to 99 degrees F and the base excess improved (Table 2). Simultaneously, the muscle rigidity, tachycardia, and tachypnea all resolved. The patient was continuously monitored and the remaining ICU course was uneventful. 

Generalized weakness and muscle soreness were reported up to 5 days afterward, but the patient fully recovered and was eventually discharged home. Genetic testing was sent to a lab for sequencing of both the RYR1 and CACNA1S genes, known to be associated with MH. After approximately one week, the results for our patient returned negative. Due to the extensive nature of the patient's surgery and financial concerns, the CHCT has yet to be performed.

## 3. Discussion

Malignant hyperthermia (MH) may be considered rare, but it is remarkably lethal if left untreated. Confirming a diagnosis requires harvesting a freshly biopsied muscle tissue sample to be tested at one of only four available centers in the United States. The obvious challenges to completing the caffeine-halothane contracture test can lead clinicians to treat even suspected cases of MH before having the opportunity to confirm the diagnosis. Even with a valid health insurance policy, some companies label the test as “experimental” and may limit or deny coverage altogether [2]. However, collecting a blood sample while in the ICU to send for MH susceptibility genetic testing was a viable option for our case. Mutations of the RYR1 and CACNA1S genes are associated with MH and 42 different RYR1 mutations and 2 CACNA1S mutations have been identified to date [5]. Per MHAUS, RYR1 gene sequencing is currently available at only two accredited molecular genetics laboratories in the United States [2] and substantial efforts were required on our part in order to link the laboratory at our hospital with one of these testing centers. Genetic testing for diseases is becoming more accessible, spurring medical advances, but is also increasing the interest of insurance companies to develop more accurate customer risk stratifications [6]. The outcome of these genetic tests could therefore affect the pricing or structure of insurance available to a patient found to have a genetic disease or abnormality. As this fear of genetic discrimination began to permeate society and leach into health policy and political agendas, legislation introducing some nondiscrimination provisions was included in the Health Insurance Portability and Accountability Act (HIPAA) of 1996 and the Genetic Information Nondiscrimination Act (GINA) of 2008 [7]. A handful of states in the United States also provide some additional protective provisions. The idea was to prevent health insurers or health plan adjusters from utilizing genetic information to determine a patient's coverage and premium adjustments or impose exclusions for preexisting conditions. GINA also prohibited the use of genetic information to make decisions regarding hiring, firing, promotion, or other terms of employment [7]. It is important to mention that GINA may have offered many protections against genetic discrimination, but several restrictions and exceptions existed allowing for loopholes with potentially profound consequences. For example, GINA provisions did not apply to life insurance, disability, or long-term care insurance, there was no mandate to provide coverage for genetic services, GINA did not prohibit the use of genetic test information in health insurance reimbursement decisions, and once genetic information has manifested itself into an actual health condition, this condition would no longer be protected by GINA [6, 7]. More important to the discussion of our patient with suspected MH, genetic testing is highly specific, but the sensitivity can be only about 50% [8]. Therefore, a negative test cannot rule out MH susceptibility and we relied more on the clinical signs and supportive laboratory results. A clinical diagnosis based on symptomatology or the caffeine-halothane contracture test is made without the utilization of genetic testing. Therefore, there are no provisions or protections from GINA for MH testing performed on an* in vitro* muscle biopsy. The recent Affordable Care Act (ACA) of 2010 and the GINA 2011 update enhanced consumer protections in the private health insurance market to prohibit issuers of health insurance from discriminating against patients with genetic diseases by refusing coverage or adjusting premiums because of preexisting conditions [7]. Our patient's RYR1/CACNA1S genetic test did not return with any identifiable mutations and the caffeine-halothane test is pending at the patient's discretion. Our decision to administer dantrolene was based on the guidance provided by the MHAUS 24-Hour Hotline and the utilization of the clinical grading scale developed by Larach and colleagues (Table 4), wherein a higher score implies a greater likelihood of a MH. Our patient received a raw score of 83 points, where a score of 50+ points is “almost certainly” associated with a diagnosis of MH [3, 9]. In further support of a probable diagnosis of MH, the rapid reversal of metabolic acidosis associated with dantrolene administration increased our patient's Larach scale score by 5 additional points to a total score of 88 [3, 9].

Pending confirmation, the patient's medical record mentions suspicion of MH susceptibility and treatment with dantrolene for malignant hyperthermia. This information will remain in the patient's permanent medical record and could be viewed as a preexisting condition. With the current protections put fourth by the ACA and GINA, a clinical diagnosis of MH should not put the patient at risk of being rejected by any health insurance plan, paying higher premiums, or result in refusal of payment while carrying a diagnosis of MH. However, the ACA does not cover life insurance, disability, or other types of supplemental insurance and our patient would continue to be at risk for wide variability of access or affordability. The current debates for repealing or replacing of any of the protections offered by the ACA or adopting the proposed American Health Care Act may completely change our patient's access to even basic health insurance in the future.

In conclusion, making a clinical diagnosis of MH can be problematic and confirmation of the disease is cumbersome and not usually possible on a single admission. There is no test that can be applied acutely to distinguish MH from other causes of hypermetabolism or hyperthermia [4]. The symptoms may occur at any time during the perioperative period and can be highly variable. This case is unique in that our patient developed suspicious symptoms at a very late stage of the perioperative period, given that the patient inhaled volatile anesthetic for 13 hours before any symptoms appeared. We stress the importance of considering MH even with an unusual or extremely late presentation and also pose that prompt, proactive treatment with dantrolene may significantly blunt the disease process. MHAUS recommends that all facilities where MH triggering anesthetics and depolarizing muscle relaxants are administered should stock dantrolene [10]. It is widely available, from generic formulations to more sophisticated preparations designed to reduce the number of vials needed for reconstitution of the drug. Brandom et al. demonstrated that the complications with dantrolene are rarely life-threatening but may include muscle weakness (14.6%), phlebitis (9.2%), and gastrointestinal upset (4.3%) [11]. The benefits of using dantrolene with our patient certainly outweighed the risks, resulting in a favorable outcome, but such prudent clinical decisions may have long-lasting effects. Pinyavat et al. explain that miscoding for MH, preemptively treating suspicious cases with dantrolene (as with our case), and even simply having a family member with the disease have contributed to a significant number of cases being added to Medicaid and Medicare databases, as well as individual state hospital discharge databases, listing MH as “present on admission,” and have resulted in MH being identified as a preexisting condition [4]. Without confirmation via the caffeine-halothane contracture test, we will never be certain of the diagnosis with our patient. This case of suspected MH, in the arena of an uncertain health insurance market, has highlighted the importance of improving the accuracy and awareness of diagnosing someone with a serious disease capable of marring the patient's permanent health record with a preexisting condition.

## Figures and Tables

**Table 1:** Arterial-blood gases at 3 and 2 hours before end of surgery.

Date	10/10/16	10/10/16
Time	17:54 (3 hours before surgery end)	19:04 (2 hours before surgery end)

Glucose (65-99 mg/dL)^a^	151	144

pH^b^ (7.35 – 7.45)	7.37	7.36

pCO2^c^ (35-45 mmHg)^d^	40.7	39.2

pO2^e^ (65-100 mmHg room air)^d^	173	177

BE^f^ (-3- + 3 mEq/L)^g^	0	-3

HCO3^h^ (22-26 mmol/L)^i^	24.3	22.6

Hb^j^ (12.0-16.0 g/dL)^k^	5.5	9.5

Hct^l^ (36-48 %)	17	28

O2Hb^m^ (>90 %)	100	100

Potassium (3.5–5.0 mmol/L)^i^	3.8	3.7

Sodium (136-145 mmol/L)^i^	138	140

^a^mg/dL = milligrams per deciliter; ^b^pH = potential of hydrogen; ^c^pCO2 = partial pressure of carbon dioxide; ^d^mmHg = millimeters of mercury; ^e^p02 = partial pressure of oxygen; ^f^BE = base excess; ^g^mEq /L = milliequivalents per liter; ^h^HCO3 = bicarbonate ion; ^i^mmol/L = millimoles per liter; ^j^Hb = hemoglobin; ^k^g/dL = grams per deciliter; ^l^Hct = Hematocrit; ^m^O2Hb = oxygen saturation of hemoglobin.

**Table 2:** Arterial-blood gases during postoperative course.

Date	10/10/16	10/10/16	10/11/16	10/11/16	10/11/16	10/11/16	10/11/16	10/11/16	10/12/16
Time	***23:07*** ^***1***^	23:15	*00:32* ^*2*^	02:20	03:07	09:35	15:30	21:22	04:45

pH^a^ (7.35 – 7.45)	***7.139***	7.179	*7.468*	7.653	7.532	7.608	7.51	7.5	7.424

pCO2^b^ (35-45 mmHg)^c^	***31.6***	31.4	*25.8*	14.4	21.7	21	26.8	28.9	36.7

pO2^d^ (65-100 mmHg)^c^	***221***	213	*157.6*	123	565.7	95.5	104.7	83	67.7

BE^e^ (-3 - + 3 mEq/L)^f^	***-17***	-15	*-4.2*	-3.9	-4.1	-0.4	-1.2	-0.1	-0.8

HCO3^g^ (22-26 mmol/L)^h^	***10.7***	11.7	*18.3*	15.6	17.8	20.6	21	22	23.5

Hb^i^ (12.0-16.0 g/dL)^j^	***9.5***	9.9	*11*	8	7.4	7.2	10.1	12.2	7.3

O2Hb^k^ (>90 %)	***100***	100	*97.9*	98	98.7	97.1	97.6	95.6	92.7

Potassium (3.5-5.2 mmol/L)^h^	***3.4***	3.5	*4.1*	3.7	3.6	3.1	3.2	3.6	3.8

Sodium (135-145 mmol/L)^h^	***140***	142	*145*	141	141	142	140	140	137

^a^pH = potential of hydrogen; ^b^pCO2 = partial pressure of carbon dioxide; ^c^mmHg = millimeters of mercury; ^d^p02 = partial pressure of oxygen; ^e^BE = base excess; ^f^mEq/L = milliequivalents per liter; ^g^HCO3 = bicarbonate ion; ^h^mmol /L = millimoles per liter; ^i^Hb = hemoglobin; ^j^g /dL = grams per deciliter; ^k^O2Hb = oxygen saturation of hemoglobin.

^**1**^ **Time of first arterial-blood gas after anesthesia consulted for suspected MH.**

^2^Time of first arterial-blood gas after treatment with dantrolene.

**Table 3:** Postoperative supporting follow-up labs from ICU.

Date	10/11/16	10/11/16	10/11/16	10/11/16	10/11/16	10/11/16	10/11/16	10/12/16
Time	0:00	2:00	2:49	5:50	9:38	15:00	20:32	4:50

Creatine Kinase (26-192 U/L)^a^	913	1038			1022	1177		

CK-MB^b^(0-6 ng/mL)^c^	9.1	7			7		3	

Myoglobin, plasma (28-58 ng/mL)^c^			888		638	566	410	261

Troponin-T (0-0.06 ng/mL)^c^	<0.01	<0.01						

Lactic Acid, plasma (0.5-2.0 mmol/L)^d^	10			3.9				

^a^U/L = units per liter; ^b^CK -MB = creatine kinase muscle/brain; ^c^ng /mL = nanograms per milliliter; ^d^mmol/L = millimoles per liter.

**Table 4:** Larach et al.'s clinical grading scale for malignant hyperthermia^1^.

**Clinical finding ** **(maximum score) **	**Manifestation **
Respiratory acidosis (15 points)	End-tidal CO_2_ >55 mmHg, PaCO_2_ >60 mmHg

Cardiac involvement (3 points)	Unexplained sinus tachycardia, ventricular tachycardia, or ventricular fibrillation

Metabolic acidosis (10 points)	Base deficit >8 mEq/L, pH <7.25

Muscle rigidity (15 points)	Generalized rigidity, severe masseter muscle rigidity

Muscle breakdown (15 points)	Serum creatine kinase concentration >20,000/L units, cola-colored urine, excess myoglobin in urine or serum, plasma [K+] >6 mEq/L

Temperature increase (15 points)	Rapidly increasing temperature, T >38.8° C

Other	Rapid reversal of MH signs with dantrolene (score=5 points), elevated resting serum creatine kinase concentration (score=10 points)

Family history (15 points)	Consistent with autosomal dominant inheritance

(1) From Rosenberg H, Sambuughin N, Riazi S, Dirksen R. Malignant hyperthermia susceptibility; synonym: malignant hyperpyrexia. 2003 [Updated 2013]. In: Adam MP, Ardinger HH, Pagon RA, et al., editors. GeneReviews [Internet]. University of Washington, Seattle; 1993-2018. Available from: https://www.ncbi.nlm.nih.gov/books/NBK1146/. Accessed October 13, 2018.
